# The Study of Soliton Mode-Locked and Bound States in Erbium-Doped Fiber Lasers Based on Cr_2_S_3_ Saturable Absorbers

**DOI:** 10.3390/ma18040864

**Published:** 2025-02-16

**Authors:** Dong Li, Ruizhan Zhai, Yongjing Wu, Minzhe Liu, Kun Zhao, Qi Yang, Youwei Dong, Xiaoying Li, Xiaoyang Wu, Zhongqing Jia

**Affiliations:** Shandong Key Laboratory of Optoelectronic Sensing Technologies/National-Local Joint Engineering Laboratory for Energy and Environment Fiber Smart Sensing Technologies, Laser Institute, Qilu University of Technology (Shandong Academy of Sciences), Jinan 250104, Chinaminzheliu@sdlaser.cn (M.L.); dongyouwei200003@163.com (Y.D.);

**Keywords:** bound-state solitons, Cr_2_S_3_ saturable absorber, soliton mode-locking

## Abstract

Femtosecond fiber lasers are widely utilized across various fields and also serve as an ideal platform for studying soliton dynamics. Bound-state solitons, as a significant soliton dynamic phenomenon, attract widespread attention and research interest because of their potential applications in high-speed optical communication, all-optical information storage, quantum computing, optical switching, and high-resolution spectroscopy. We investigate the effects of pump power variations on the formation of mode-locked solitons and bound-state solitons in a femtosecond fiber laser with a Cr_2_S_3_ saturable absorber (SA) through numerical simulations while observing the transition, formation, and break-up process of bound soliton pulses. By optimizing the cavity structure and adjusting the net dispersion, the mode-locked soliton is obtained based on this SA. This is the narrowest solitons produced by this SA to date, exhibiting the smallest time-bandwidth product. Moreover, stable double-bound solitons and unique (2 + 1) triple-bound solitons are successfully obtained. The diverse bound-state solitons not only demonstrate the excellent nonlinear absorption properties of Cr_2_S_3_ as a saturable absorber but also expand the scope of applications for Cr_2_S_3_ saturable absorbers in fiber lasers.

## 1. Introduction

Femtosecond fiber lasers, with their ultra-high peak power density, extremely short pulse duration, broad spectrum, excellent environmental stability [1], etc., play an important role in fields such as communication [2], military [3], and medical treatment [4], etc. As a typical nonlinear dissipative system, erbium-doped femtosecond fiber lasers have also become an ideal platform for exploring soliton dynamics [5], facilitating the observation of a wide range of soliton phenomena. Fibers typically have a limited mode field area. When the peak power reaches a certain threshold, due to the soliton area theory and the quantization effect of pulse energy [6], laser pulses undergo soliton break-up and interact to form higher-order solitons. Among these, bound-state solitons represent a distinct manifestation of higher-order solitons, comprising soliton bundles formed by two or more sub-pulses that are coupled through nonlinear interactions. These sub-pulses exhibit mutual attraction when they deviate significantly from their equilibrium distance, and conversely, they repel each other when they come closer [7]. The temporal spacing between adjacent sub-pulses plays a pivotal role in the formation of bound-state solitons [8]. This parameter allows for the classification of bound states into two categories: loosely bound [9] and tightly bound states [10]. Loosely bound states refer to configurations where the spacing between adjacent solitons exceeds several pulse widths. Additionally, it has been found that the soliton pairs can also act as a unit to form other bound states of solitons, such as the (2 + 2), (2 + 1), and (2 + 2 + 2) configurations [11]. Currently, bound states have been utilized to improve low-order harmonics and for the generation of higher-order optical harmonics in the design of photonic devices. By applying bound states, light-matter interactions hold the potential for designing solid-state attosecond spectroscopy and ultraviolet source technologies. Furthermore, through bound states, quantum confinement effects are being explored to leverage optical properties for applications in quantum information processing, particularly in the design of qubits and nonlinear quantum computations [12].

Despite the considerable attention and extensive studies on bound-state solitons, their regulation remains challenging in conventional optical media, primarily due to the limited availability of tunable system parameters [13]. To address this, researchers have explored various approaches to control the dynamics of bound-state solitons. For example, Nguyen et al. employed periodic phase modulation to generate phase chirp of the generated lightwaves in the ring laser. Their work revealed that the chirped phase state not only facilitates the formation of multi-soliton-bound states but also enhances their stability [14]. Qin et al. utilized the Rydberg-EIT system to generate nonlocal Kerr nonlinear effects, successful generation, and manipulation of high-dimensional weak-light soliton molecules [13]. While various approaches have been explored for the formation and control of intracavity bound solitons, the use of SAs with exceptional properties offers a more convenient method for achieving bound-state soliton regulation [15]. For example, Liu et al. utilized a carbon nanotube-based SA in a fiber laser and achieved dual-soliton states with discrete equilibrium distances by employing narrowband filtering through a fiber Bragg grating [16]. Similarly, Wang et al. implemented VSe_2_/GO SA in a fiber laser and successfully generated soliton molecules by modulating the polarization controller and pump power [17]. Nonetheless, studies focusing on soliton control through SAs and pump power remain limited.

**Figure 1:**
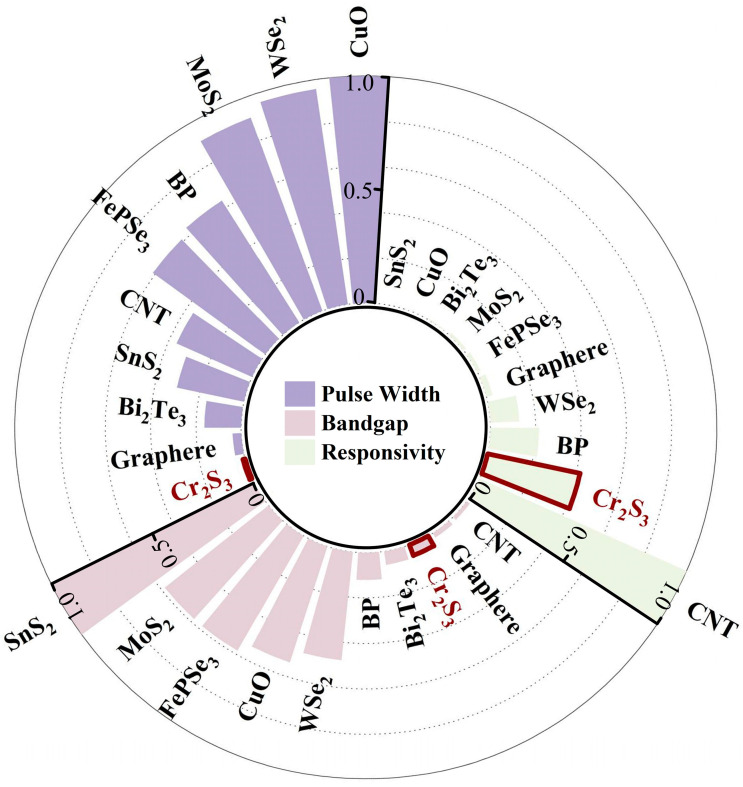
Comparison of commonly used saturable absorbers in terms of bandgap energy (pink), responsivity (green), and mode-locked pulse width (purple). All parameters are normalized for clarity. Data for bandgap energy and responsivity are referenced from [23,25,26,27,28,29,30,31,32,33], while mode-locked pulse widths are compiled from [34,35,36,37,38,39,40,41,42]. The three characteristics of the materials in this study are labeled in the figure.

In recent years, chromium disulfide (Cr_2_S_3_), as a member of transition metal chalcogenides, has garnered significant attention due to its exceptional nonlinear optical properties [18]. At the same time, the application of Cr_2_S_3_ in the field of lasers has also been demonstrated, showing outstanding performance [19,20]. Its nonlinear photoresponses exhibit minimal dependence on film thickness variations [21] and hold great potential for the design of advanced optoelectronic devices, such as saturable absorbers [20]. Compared to other two-dimensional materials, such as black phosphorus, Cr_2_S_3_ offers excellent optical stability in ambient environments [22]. We compare three key characteristics of typical two-dimensional materials’ SAs: bandgap, responsivity, and the pulse width of mode-locked lasers based on these SAs, as shown in Figure 1. In this figure, the parameters of the three characteristics are normalized, and an axial coordinate system is established. The relative magnitude of each parameter is represented by the length of the corresponding sector component. In terms of responsivity, materials with higher response rates demonstrate the potential to achieve mode-locking under weaker optical signals. Cr_2_S_3_ demonstrates a response rate of 3 A·W^−1^ under ambient conditions at a central wavelength of 1550 nm [22], second only to carbon nanotubes, which have a response rate of 7.2 A·W^−1^ [23], and significantly outperform other commonly used materials such as Graphene, SnS_2_, and Bi_2_Te_3_, etc. The bandgap of a material significantly influences the wavelength and range of mode-locked solitons. Cr_2_S_3_, with a direct bandgap of approximately 0.15 eV [22], fills the gap between the near-zero bandgap of single-walled carbon nanotubes and the relatively large bandgaps of transition metal dichalcogenides [24]. This property endows Cr_2_S_3_ with exceptional and highly efficient saturable absorption capabilities in the 1.5 μm (0.83 eV) wavelength region, supporting ultrashort femtosecond pulse durations [20]. In terms of soliton mode-locking pulse width, Yang et al. successfully fabricated a Cr_2_S_3_-based SA in 2022 and observed traditional soliton mode-locking as well as triple-pulse bound soliton operation in an erbium-doped fiber laser. The pulse width and time-bandwidth product (TBP) were measured to be 4.60 ps and 2.36, respectively, due to the presence of a large frequency chirp in the mode-locked soliton [20]. In 2024, Qiao et al. investigated Cr_2_S_3_-based Q-switched and mode-locked fiber lasers, reporting a mode-locked pulse with a duration of 10.2 ns [19]. A comparison of the mode-locked pulse widths of several saturable absorbers reveals that the Cr_2_S_3_ SA in this study generates a mode-locking pulse width of 329 fs, which is significantly shorter than that of most other saturable absorbers, reflecting the superior performance of this SA. These findings underscore the exceptional potential of Cr_2_S_3_ in generating femtosecond lasers and regulating soliton-bound states. However, there is limited research on this saturable absorber in passive mode-locked fiber lasers. It is highly significant to deeply explore the formation and evolution of mode-locked solitons and bound solitons in femtosecond fiber lasers based on Cr_2_S_3_ SA through numerical simulations.

In this work, a physical model for ultrafast soliton evolution based on Cr_2_S_3_ SA was established. By designing and optimizing the cavity structure, femtosecond-level mode-locked pulses were theoretically achieved, and a wealth of bound soliton phenomena was observed. Based solely on the exceptional characteristics of the SA and the adjustment of the intracavity gain, mode-locked solitons with a pulse width of 329 fs were achieved in the simulation, with the corresponding TBP of 0.332. Using the designed cavity structure, multi-bound soliton evolution was realized by controlling the small-signal gain coefficient proportional to the pump power, leading to the formation of double-bound solitons and unique (2 + 1) triple-bound solitons. Numerical simulations reveal the evolution process of bound solitons, systematically simulating and analyzing the transitional evolution between different soliton states, thereby theoretically validating the feasibility of this regulation method.

## 2. Numerical Model of Erbium-Doped Fiber Laser Based on Cr_2_S_3_-SA

To simulate the soliton evolution within a fiber laser, the numerical cavity model is established based on the modified nonlinear Schrödinger equation:

(1) $$\frac{\partial A}{\partial z}+\frac{i}{2}(\beta_{2}+ig{T_{2}}^{2})\frac{\partial^{2}A}{\partial T^{2}}=\frac{1}{2}(g-\alpha )A+i\gamma {\left|A\right|}^{2}A$$

In this equation, *A* is the electric envelope of the pulse, *β*_2_ represents the second-order dispersion parameter of the fiber, *α* corresponds to the attenuation coefficient, and *γ* is the nonlinear coefficient. The pulse propagation in the cavity is solved using the split-step Fourier method [43]. Under the action of the intracavity gain and the saturable absorption effect caused by the nonlinear device, the dominant pulse, that is, the soliton, gradually forms. The parameter *T*_2_, associated with the gain bandwidth (Ω*_g_*) of the gain fiber, is defined as follows:

(2) $$T_{2}=1/\Omega_{g}$$

Considering the gain spectrum range of erbium-doped fiber (EDF), Ω*_g_* is set to 40 nm [44]. The nonlinear coefficient *γ* is defined as follows:

(3) $$\gamma =\omega_{0}n_{2}/(cA_{\mathrm{eff}})$$

where *ω*_0_ is the central frequency, *n*_2_ is the nonlinear refractive index, *c* is the speed of light, and *A*_eff_ is the effective mode area. The saturated gain coefficient of the active fiber, *g* is calculated [45] as follows:

(4) $$g=\frac{g_{0}}{1+\frac{E_{0}}{E_{\mathrm{sg}}}}$$

Here, *E*_0_ is intracavity pulse energy, *E*_sg_ corresponds to the gain saturation energy, with a model value of 1.4 nJ, and *g*_0_ represents the small-signal gain coefficient determined by the pump power, expressed as follows:

(5) $$g_{0}=P_{p}A=P_{p}\frac{\Gamma_{s}\sigma_{s}\Gamma_{p}\sigma_{p}}{A_{\mathrm{eff}}hv_{p}}$$

In this expression, Γ_s_ and Γ_p_ are the overlap factors for the signal and pump light, respectively, σ_s_ and σ_p_ denote the cross-sections at the transition frequencies of the signal and pump light, *P*_p_ is the pump power, *h* is Planck’s constant, and *v*_p_ is the pump frequency. Consequently, the small-signal gain coefficient is directly proportional to the *P*_p_. *E*_0_ is computed as follows:

(6) $$E_{0}=\int {\left|A\right|}^{2}dT$$

As an essential component in mode-locked femtosecond fiber lasers, the SA exhibits a decreasing absorption coefficient as the light intensity increases. The transmission rate *t*_1_ of the SA to the light field is modeled by the following fitted equation [46]:

(7) $$t_{1}=1-\frac{\Delta T}{1+\left(\frac{E_{0}}{E_{s}}\right)}-\alpha_{\mathrm{ns}}$$

In the equation, Δ*T* is the modulation depth of the SA, *E*_s_ is the saturation intensity of the SA, and *α*_ns_ denotes the nonsaturable loss. To make the Cr_2_S_3_ SA used in the model more realistic, the modulation depth, nonsaturable loss, and saturation intensity in the model are set based on the data measured in [20], which are 6.36%, 34.99%, and 10.24 MW/cm^2^, respectively.

At the initial stage of mode-locked fiber laser operation, the intracavity optical signal is relatively weak and dominated by high-frequency noise, containing multiple frequency components. Thus, in the simulation, the initial pulse is modeled as a Gaussian pulse with random noise. Since achieving mode-locking requires synchronization of a single transverse mode and multiple longitudinal modes within the cavity, the amplitudes and phases of the longitudinal modes are initially random. Consequently, this leads to a relatively oscillating and chaotic state within the cavity. Based on this analysis, a Gaussian pulse with random noise is employed as the initial signal input for cavity simulations. As light circulates within the cavity, the stronger light can be amplified by the SA, and the weaker light is absorbed by the SA. Eventually, the interplay of dispersion, self-phase modulation (SPM), and gain and loss mechanisms drive the fiber laser to evolve from an initial unstable Gaussian noise pulse into a stable mode-locked pulse.

The initial Gaussian pulse with random noise can be expressed as follows:

(8) $$A(t)=\sqrt{P_{0}}\left[NLrand(1,M)+e^{-\frac{1}{2}(1+iC_{0}){\left(t/T_{0}\right)}^{2}}\right]$$

where *P*_0_ is the peak power, *NL* is the noise level, *M* is the number of data points, *T*_0_ is the pulsewidth, and *C*_0_ is the chirp of the initial Gaussian pulse. The initial value of those parameters is shown in Table 1, including the time-domain window *T*_max_, the central wavelength *λ*_0_, and the pulse full-width of half maximum (*T*_FWHM_).

The central wavelength is selected as 1550 nm due to the higher gain coefficient and broader gain bandwidth of erbium-doped fibers at this wavelength, which enables efficient amplification of optical signals. These characteristics promote the generation and stable propagation of mode-locked solitons and bound-state solitons. Additionally, the 1550 nm wavelength lies within the low-loss window of fibers, rendering it particularly advantageous for optical communication systems [47]. The relationship between *T*_FWHM_ and *T*_0_ is expressed as follows:

(9) $$T_{0}=\frac{T_{\mathrm{FWHM}}}{2\sqrt{\ln\left(2\right)}}$$

The parameter *NL* can be adjusted between 0 and 1 without impacting the simulation results [48]. Additionally, the *T*_FWHM_ of the initial noise is set to 10 ps because the initial noise only plays a role in the early stage, while the characteristics of the final stable mode-locked pulse are determined by the interplay of gain, loss, SPM, and dispersion during cavity evolution. As such, variations in the *T*_FWHM_ within a reasonable range do not affect the simulation outcomes. Moreover, 10 ps serves as a threshold between wide-pulse and narrow-pulse evolution, enabling the evaluation of the model’s tendency to transition toward narrow pulses or experience pulse break-up under varying conditions, thus facilitating the analysis of the nonlinear optical system’s stability [49].

**Figure 2:**
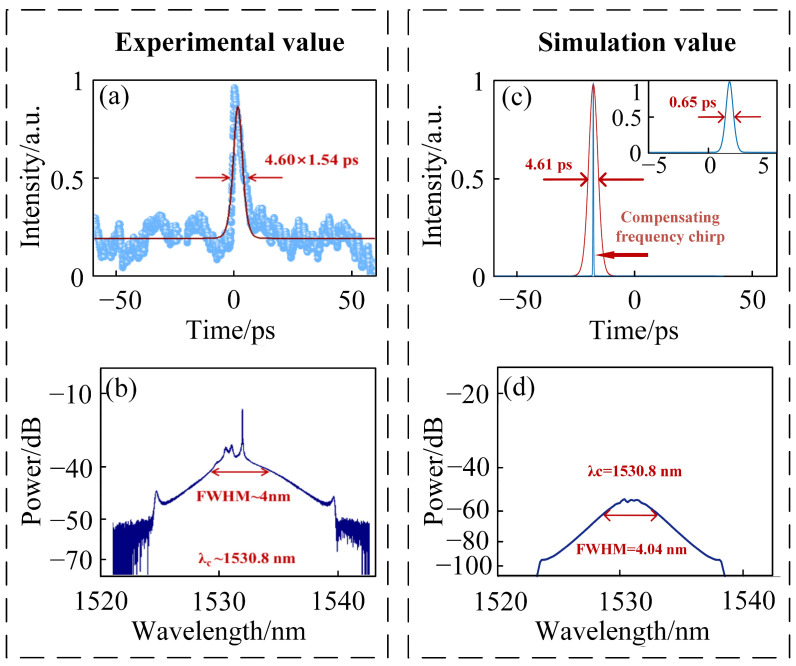
(**a**) Experimentally obtained mode-locked soliton pulse width and (**b**) spectral bandwidth [20]; (**c**) simulated mode-locked soliton pulse width and (**d**) spectral bandwidth.

To evaluate the accuracy of the proposed model, a corresponding simulation framework is constructed based on the experimental setup, fiber parameters, and central wavelength reported by Yang et al. [20]. A comparison between the experimental and simulated results was performed. As shown in Figure 2a,b, the mode-locked soliton observed in the experiment exhibited a pulse width of 4.60 ps and a spectral bandwidth of approximately 4 nm, yielding a TBP of 2.36. The simulated results under the corresponding parameters, presented in Figure 2c,d, reveal mode-locked solitons with a pulse width of 4.61 ps and a spectral bandwidth of 4.04 nm. The comparison in Figure 2 demonstrates excellent agreement between the experimental and simulation results, thereby validating the accuracy of the proposed model. This model can thus serve as a reliable tool for investigating pulse evolution dynamics in fiber lasers.

In this simulation, the mode-locked pulse exhibits a pulse width of 4.61 ps and a corresponding spectral bandwidth of 4.04 nm, indicating significant frequency chirp accumulation within the pulse. Through calculation, the chirp amount within the pulse is determined to be 7.49 [43]. Furthermore, by compensating for the intra-cavity chirp, the final mode-locked pulse width achieved is 0.65 ps, as illustrated in the inset of Figure 2c. The corresponding TBP is 0.336, which is close to the theoretical transform-limited value.

**Figure 3:**
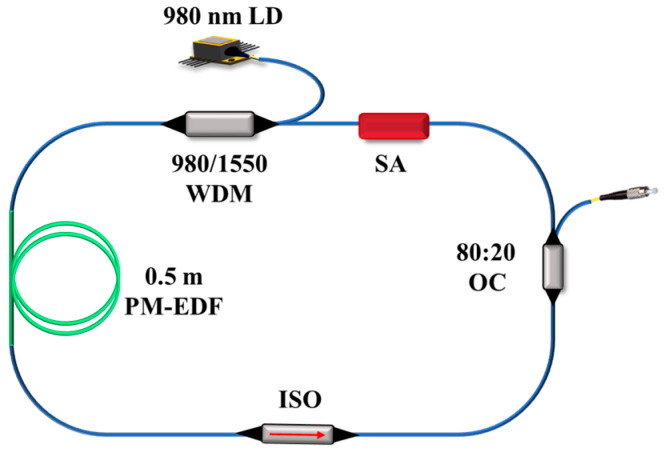
Schematic diagram of the fiber laser model based on Cr_2_S_3_ SA.

The introduction of substantial net negative dispersion within the cavity results in a relatively broad mode-locked soliton pulse. Further reduction of the net dispersion in the cavity is advantageous for narrowing the pulse width of mode-locked solitons. Based on this premise, a laser simulation model is designed, as illustrated in Figure 3. A 0.5 m high-doped polarization-maintaining EDF serves as the gain medium, with a 980 nm laser diode providing the pump source. The pump light is coupled into the laser via a 980/1550 nm wavelength division multiplexer. The cavity is constructed entirely with polarization-maintaining fiber components. After passing through a fiber isolator to ensure unidirectional pulse propagation, the intracavity signal with a central wavelength of 1550 nm is extracted via an 80:20 coupler. Most of the laser is directed through the SA for pulse modulation based on its saturable absorption characteristics, while a small portion is output through the coupler to complete the fiber laser ring cavity.

Table 2 summarizes the parameters of the active fiber and passive single-mode fiber (SMF) used in the simulation. The total length of the SMF is set to 2.05 m, achieving a net dispersion of −0.035 ps^2^ within the cavity. The entire cavity length is 2.55 m. The pulse repetition frequency in the cavity is determined by the following formula [7]:

(10) $$f=\frac{c}{nL}$$

where *n* is the refractive index of the fiber, and *L* denotes the total cavity length, corresponding to a fundamental repetition frequency of 80.5 MHz. Since the pulse width is typically related to the recovery time of the saturable absorber, and the observed soliton pulse width of 4.60 ps is consistent with a fast recovery time [20], we infer that the recovery time of the Cr_2_S_3_ SA used in our system is also on the ps scale.

The pulse evolution process in mode-locked fiber lasers is highly dependent on the pump power. In this numerical model, when the pump power-related value *g*_0_ increases, the nonlinear effects within the cavity become prominent. When *g*_0_ reaches the mode-locking threshold, the laser cavity transitions into a stable mode-locked state under the combined influence of dispersion and SPM.

Theoretically, increasing the pump power can generate additional bound states of solitons, attributed to the interplay between the soliton area theorem and pulse energy quantization effects. Soliton break-up only occurs when the pump power reaches a sufficiently high level [50]. The soliton area formula is given by the following [47]:

(11) $$A_{0}T_{\mathrm{FWHM}}=\sqrt{\frac{2\left|D\right|}{\delta }}$$

In the soliton area equation, *A*_0_ represents the peak amplitude of the pulse, *D* denotes the net dispersion within the cavity, and *δ* accounts for the contribution of SPM in the Kerr medium.

Once the optical cavity is established, parameters such as dispersion and nonlinearity in the equation remain constant, resulting in a fixed product of pulse peak amplitude and pulse duration. As is commonly known, increasing the pump power results in a reduction in pulse width. However, constrained by the relaxation time of the Cr_2_S_3_ SA, the pulse width ceases to decrease further once the limit is reached. At this stage, if *g*_0_ is further increased, the peak amplitude rises. According to the soliton area Formula (11), the peak amplitude cannot increase indefinitely, resulting in soliton break-up. This phenomenon is validated later in the text.

## 3. Numerical Simulations and Results

### 3.1. Soliton Mode-Locking

Based on the constructed physical model and the parameters of the Cr_2_S_3_ SA, an optimized cavity design, as depicted in Figure 3, is developed to maintain a net dispersion of −0.035 ps^2^ within the cavity. By controlling the intracavity gain, the rich nonlinear effects are adjusted, thereby revealing the pulse evolution within the cavity. The pulse evolution process in the fiber laser is characterized in terms of roundtrip time and the number of roundtrips.

**Figure 4:**
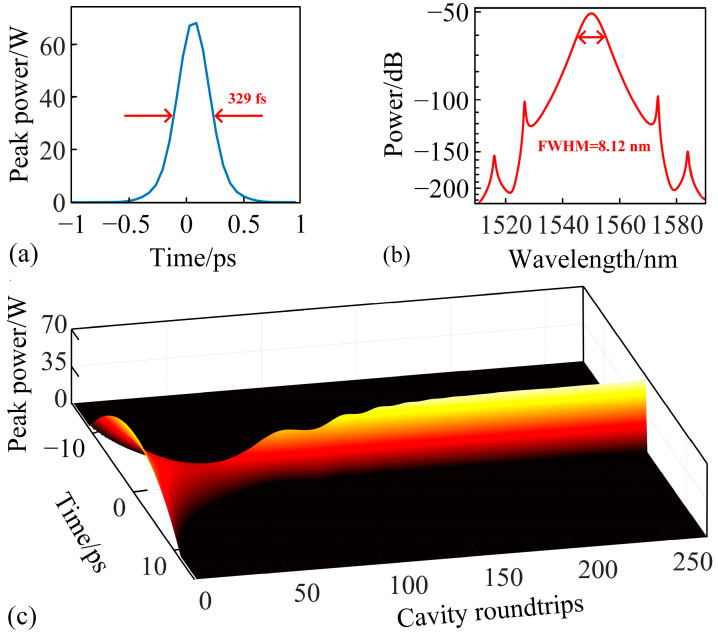
(**a**) Temporal profile of the mode-locked pulse at *g*_0_ = 1.630 after achieving stable mode-locking; (**b**) corresponding optical spectrum of the mode-locked soliton; (**c**) real-time evolution of the soliton from initial noise to stable mode-locking state.

By incrementally increasing the small-signal gain coefficient in steps of 0.001 to simulate an increase in pump power, a stable mode-locked state is achieved when the small-signal gain coefficient reaches 1.630. The resulting pulse width, as shown in Figure 4a, is determined to be 329 fs via sech-shaped fitting. The corresponding 3 dB spectral bandwidth is measured at 8.12 nm, with Kelly sidebands appearing on both sides of the central wavelength, as illustrated in Figure 4b. These features are characteristic of soliton mode-locking, confirming the generation of the mode-locked pulse within the cavity. The calculated TBP of the mode-locked soliton is 0.332, closely approaching the theoretical transform-limited value, indicating that the soliton exhibited only minimal nonlinear chirp. The real-time evolution of the soliton is depicted in Figure 4c. As seen in the figure, mode-locking commences after approximately 70 cavity roundtrips. However, due to the imbalance between gain and loss within the cavity, the peak power of the mode-locked pulse exhibits oscillations. After 160 roundtrips, the pulse intensity gradually stabilizes and remains constant, reflecting the establishment of a stable mode-locked state.

**Figure 5:**
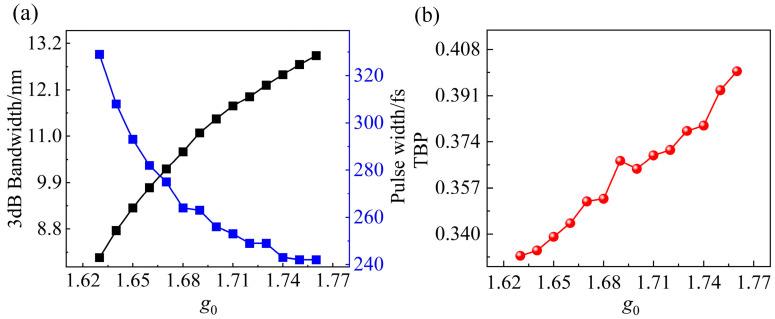
(**a**) Variation of the 3 dB spectral bandwidth (black) and mode-locked pulse width (blue) as a function of the small-signal gain coefficient *g*_0_; (**b**) evolution of TBP with *g*_0_. As *g*_0_ increases, the TBP deviates from the transform-limited value, reflecting the impact of gain narrowing on pulse quality.

By further increasing the pump power, the intracavity optical intensity rises, enhancing the nonlinear effects, particularly SPM, which is proportional to the optical intensity. This enhancement of SPM generates additional frequency components, leading to spectral broadening, as illustrated in Figure 5a. As the spectrum broadens, the mode-locked pulse width correspondingly decreases. However, due to the relaxation time limitation of the Cr_2_S_3_ SA, the pulse width stabilizes at 242 fs when *g*_0_ = 1.750. The increased nonlinear effects within the mode-locked soliton result in the TBP deviating progressively from the theoretical transform-limited value. This indicates an increase in the nonlinear chirp of the mode-locked soliton, leading to a gradual destabilization of the soliton. The relationship between the TBP of the soliton and the small-signal gain coefficient *g*_0_ is shown in Figure 5b.

### 3.2. Soliton Transition Phase

As the gain coefficient gradually increases, the cumulative intracavity nonlinearity, combined with pulse shaping by the SA and modulation by the soliton area theorem [6], leads to the break-up of a single soliton into multiple solitons. Stronger gain amplifies small signals, such as background noise, into sub-pulses, causing the pulse to split into double solitons or multiple solitons. During propagation within the cavity, the break-up solitons experience attractive and repulsive forces that achieve a state of dynamic equilibrium. In summary, the critical factors for soliton break up into high-order bound solitons include the amplification of small signals, sufficiently strong gain, and the balance of inter-soliton interaction forces. These conditions collectively enable the formation and stabilization of high-order bound solitons within the laser cavity.

**Figure 6:**
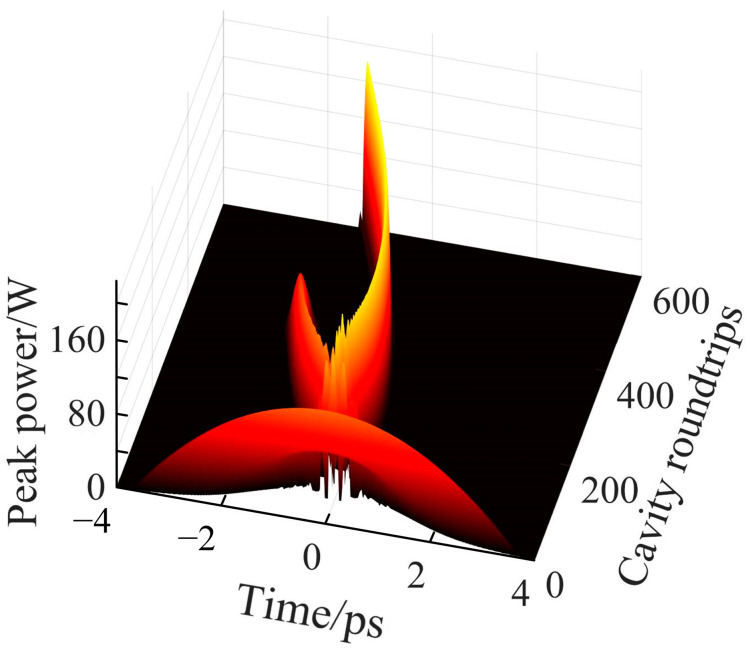
Evolution of the pulse profile during the transition stage at *g*_0_ = 1.765. This plot depicts the temporal dynamics of the peak power as a function of cavity roundtrips, highlighting the complex processes of pulse reshaping and stabilization.

During the transition from a single soliton to double solitons, a soliton transition phase is observed, as depicted in Figure 6. This phenomenon has also been reported in previous studies [51]. Figure 6 illustrates the pulse evolution during the transitional stage under a pump parameter of *g*_0_ = 1.765. Initially, the pulse undergoes continuous reshaping while emitting dispersive waves into the background. These dispersive waves can either be amplified or attenuated, depending on the strength of the gain. Once amplified to a sufficiently high level, the pulse experiences break-up, leading to the formation of multiple pulses. After the pulse breaks up, the available gain becomes insufficient to sustain two solitons simultaneously. Under the influence of gain competition [52], the soliton with higher energy secures a greater share of the gain, while the weaker soliton is progressively attenuated and eventually dissipates. As a result, only a single soliton remains. With further cavity roundtrips, the system gradually stabilizes into a steady-state mode-locked operation. In reference [53], the transition from a single pulse to a double pulse phase was achieved under near-zero dispersion conditions, eventually leading to a unique breathing phase. The transition phase in this study ultimately resulted in single soliton mode-locking, which differs from the observations in [53]. This highlights that the transition phases of higher-order solitons vary under different dispersion conditions.

### 3.3. Double-Bound Solitons

As the small-signal gain coefficient *g*_0_ gradually exceeds 1.765, the intracavity gain becomes sufficiently strong to induce soliton to break up into double solitons. However, the spacing between the two solitons remains unstable. This instability arises from an imbalance between the nonlinear and dispersive interaction forces. Additionally, the two solitons perturb each other, resulting in continuous modulation of the oscillation period and variations in their temporal separation.

**Figure 7:**
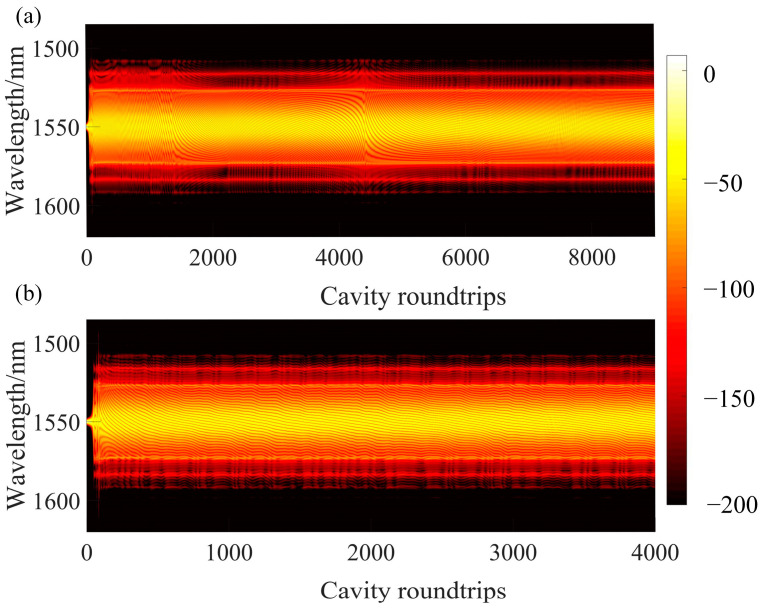
(**a**) Evolution of the unstable dual-soliton spectrum at *g*_0_ = 1.770. The horizontal axis represents the wavelength (nm), and the vertical axis denotes cavity roundtrips. The spectrum evolves within the 1500–1600 nm range over time, exhibiting instability. (**b**) Evolution of the stable double-bound solitons spectrum at *g*_0_ = 1.780. The horizontal axis represents the wavelength (nm), and the vertical axis denotes cavity roundtrips. The spectrum evolves within the 1500–1600 nm range, with the movement of fringe patterns indicating changes in the relative phase, demonstrating the stable characteristics of double-bound solitons.

Figure 7a shows the spectral evolution when *g*_0_ = 1.770. Evident interference fringes in the soliton spectrum can be observed, caused by the interference between the two pulses. After 4500 cavity roundtrips, the soliton pair remains unstable. The interference fringes progressively shift toward longer wavelengths, reflecting continuous sliding of the relative phase between the solitons. This persistent instability indicates that the balance between attractive and repulsive forces between the solitons has not yet been achieved, leaving the system in a state of dynamic adjustment. This finding underscores the complex interplay of nonlinear and dispersive effects in governing the evolution of soliton-bound states.

**Figure 8:**
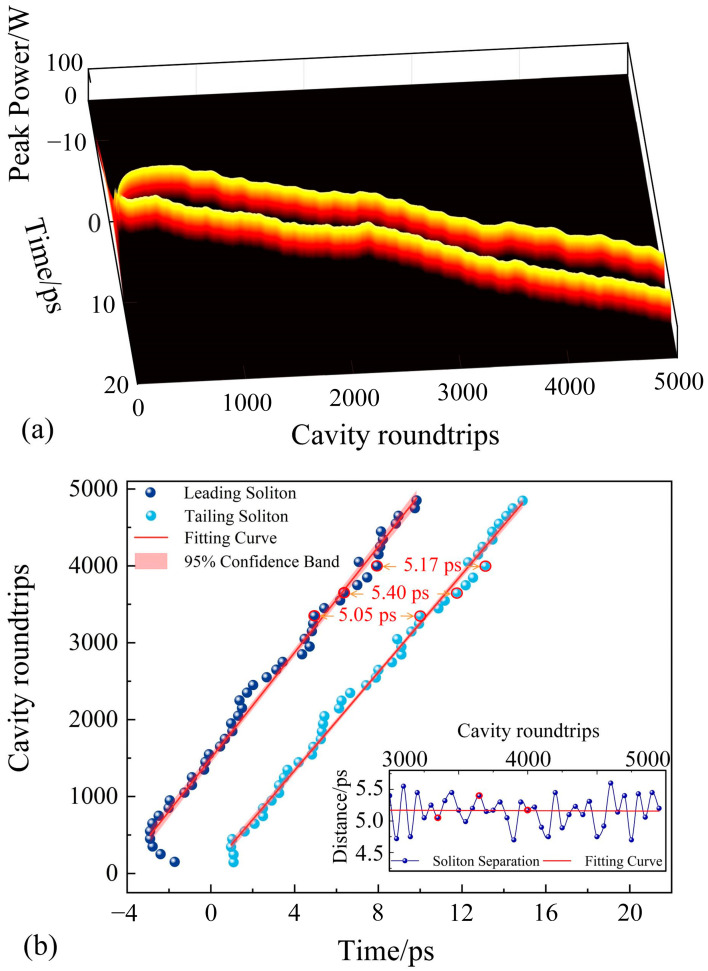
(**a**) Evolution diagram of double-bound solitons at *g*_0_ = 1.780; (**b**) sampling diagram of the spacing between double-bound solitons corresponding to (**a**) (fitted curve based on sampled points). The illustration shows the distance between the two pulses within the double-bound solitons. The red circles correspond to the three sampled points in (**b**).

As the intracavity gain continues to increase, the nonlinear attraction between solitons strengthens, gradually balancing the dispersive repulsion between pulses. When the small-signal gain coefficient *g*_0_ = 1.780, the spacing in the soliton pair approaches stability. Due to the effects of dispersion, the spectral evolution, as shown in Figure 7b, exhibits relatively smooth interference fringe variations, reflecting the continuous adjustment of the relative phase between the solitons [54]. After multiple cavity roundtrips, a stable double-bound soliton eventually forms, as illustrated in Figure 8a.

**Figure 9:**
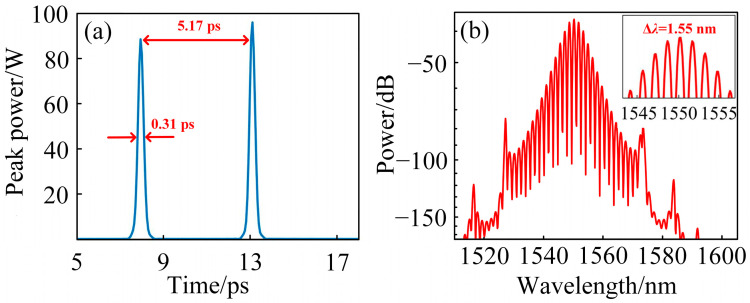
(**a**) Temporal profile of double-bound solitons after 4000 cavity roundtrips at *g*_0_ = 1.780; (**b**) spectral profile of the double-bound solitons corresponding to (**a**).

To quantitatively analyze the evolution of soliton spacing during cavity propagation, the separation between the two solitons is measured every 100 cavity roundtrips, and the results are fitted to produce a curve, as shown in Figure 8b. The horizontal axis represents the bound soliton separation, while the vertical axis denotes the number of cavity roundtrips. When the number of cavity roundtrips reaches 4000, the intracavity soliton evolution stabilizes, resulting in the pulse envelopes shown in Figure 9a. The stabilized soliton separation time is approximately 5.17 ps, with each individual soliton having a pulse width of 0.31 ps. Since the soliton separation is approximately 17 times the width of a single soliton, the system achieves a second-order loosely bound soliton state.

As shown in Figure 8b, at 3350 cavity roundtrips, the soliton separation is 5.05 ps, which is relatively small. Under these conditions, the solitons repel each other, leading to an increase in their separation. At 3650 roundtrips, the soliton separation reaches a maximum of 5.40 ps. For this relatively large separation, the solitons attract each other, causing the separation to decrease. By 4000 roundtrips, the pulse spacing stabilizes at 5.17 ps. In all cases, the soliton pair adjusts toward the equilibrium distance, signifying the formation of a stable double-bound soliton state [55]. From the fitted curve, it is evident that once the soliton pair stabilizes, the net negative dispersion within the laser cavity induces periodic interactions between the solitons and the cavity dispersion. These interactions result in periodic perturbations of the solitons, maintaining a dynamic equilibrium in the spacing between the soliton pair. The intracavity pulse evolution is extended to 5000 cavity roundtrips to analyze the fluctuation range of the separation between loosely bound solitons. As shown in the illustration of Figure 8b, the results indicate that the soliton separation fluctuations remained within a range of ±0.5 ps. This demonstrates that the system exhibits satisfactory stability. The result in this paper is similar to that reported in reference [56], which demonstrated quasi-periodic pulsating pure-quartic solitons (PQS) molecules. The quasi-periodic pulsation feature of the PQS molecules is characterized by small oscillations of ±0.4 ps around a distance of approximately 12.5 ps between two pulses. In contrast, the bound soliton pulse spacing in this study is even smaller, leading to stronger interactions.

In the spectral profile shown in Figure 9b, Kelly sidebands are still evident. These sidebands arise when the small-amplitude dispersive waves emitted by the pulse satisfy the phase-matching condition with the cavity’s free spectral range. Dispersive waves are generated as the pulse undergoes periodic gain and loss perturbations during cavity circulation, leading to periodic self-reshaping and energy release.

Dispersive waves can mediate periodic interactions between solitons based on their initial pulse separation. While other mechanisms, such as laser gain recovery and acoustic effects, have also been reported to introduce weak soliton interactions within lasers, dispersive-wave-mediated soliton interactions are the most significant [57]. These interactions play a critical role in the formation of loosely bound soliton states in lasers. In simple terms, dispersive waves influence the interactions between solitons, linking their phase and spacing. Compared to scenarios without dispersive waves, solitons can interact over relatively larger distances [54].

From the spectrum in Figure 9b, distinct modulation patterns are evident, and the pulse temporal intervals remain relatively constant. According to Fourier transform theory, the spectral modulation (Δ*λ*) of bound-state mode-locked pulses and the pulse time interval (Δ*T*) satisfy the following equation [6]:

(12) $$\Delta T=\frac{\lambda_{0}^{2}}{c\Delta \lambda }$$

By measuring the spectral modulation period as 1.55 nm, the corresponding pulse interval is calculated to be 5.17 ps using this equation. This result aligns well with the pulse interval measured in the simulation shown in Figure 9a, further confirming that the observed dual pulses correspond to double-bound solitons.

### 3.4. Triple-Bound Solitons

By gradually increasing the gain coefficient *g*_0_ in steps of 0.001, the nonlinear effects within the cavity further intensify. During this process, the evolution of solitons displays transitional states similar to those observed in the soliton transition phase described in Section 3.2. At *g*_0_ = 1.930, the solitons transition from an initial three-pulse configuration to a two-pulse configuration, indicating that *g*_0_ = 1.930 serves as the threshold for the generation of three solitons. This observation also demonstrates that the soliton transition process is not limited to the transformation from a single soliton to a two solitons state but may occur at each step of forming higher-order solitons.

**Figure 10:**
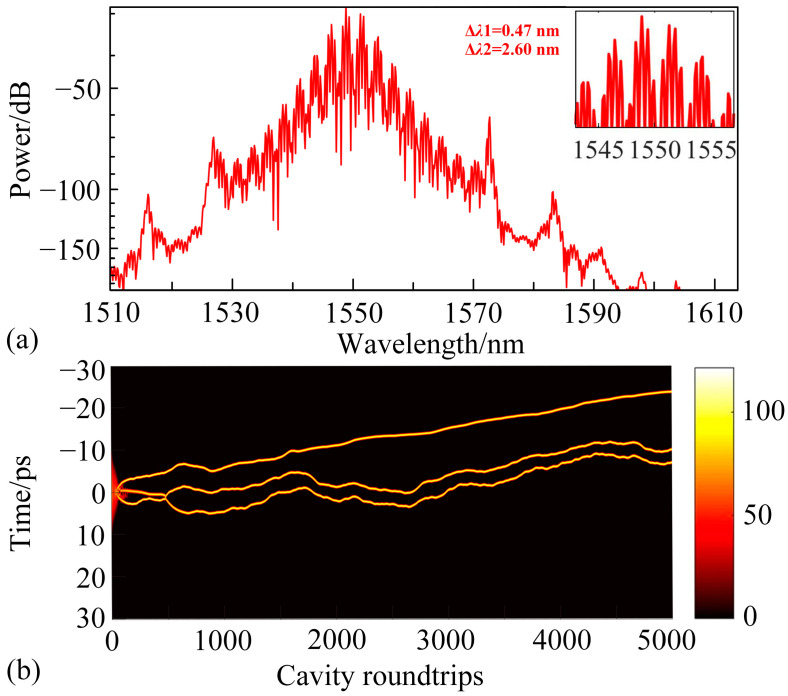
(**a**) Spectrum of the triple-bound solitons after 5000 cavity roundtrips at *g*_0_ = 1.940; (**b**) Pulse evolution of the triple-bound solitons at *g*_0_ = 1.940.

As the pump power continues to increase, the spectrum exhibits pronounced double interference fringes at *g*_0_ = 1.940, as shown in Figure 10a. With the stabilization of the solitons, a unique triple-bound solitons are observed [11]. Unlike the conventional triple-bound solitons with equal pulse intervals, this configuration features unequal spacing between the solitons, as illustrated in Figure 10b. In this figure, the horizontal axis represents the number of cavity roundtrips, while the vertical axis denotes the temporal intervals between solitons.

This bound state consists of a soliton pair and a single soliton, with the interval between the soliton pair remaining constant after stabilization. This is due to the strong nonlinear interaction between the closely spaced pulses, which allows the bound soliton pair to stably propagate as a single unit. Considering that dispersive waves induce positional oscillations of individual solitons, this is also the reason for the random fluctuations in the positions of the soliton pair, corresponding to periodic jitter. From the spectral plot of this bound state, it is observed that the spectrum undergoes dual modulation with spectral periods of Δ*λ*1 = 0.47 nm and Δ*λ*2 = 2.60 nm, respectively. The modulation period Δ*λ*1 = 0.47 nm varies within each pulse cycle, resulting in changes in the interval between the single soliton and the soliton pair. However, the modulation period Δ*λ*2 = 2.60 nm remains constant throughout the pulse cycle, leading to the formation of a 2 + 1 type triple-bound solitons.

From the pulse evolution diagram of the bound solitons, it is evident that the distances between the three solitons are unequal. The possible reasons for this can be attributed to two factors. On the one hand, the solitons in the fiber laser undergo periodic amplification, loss, and output, which may result in different interactions between the three solitons. On the other hand, as the solitons propagate in the fiber laser, dispersive waves are generated, and the interaction between the dispersive waves and the solitons also occurs within the laser cavity. However, due to the periodic effects in the fiber laser, the interactions between the solitons and the dispersive waves may differ, leading to unequal spacing between the three coupled solitons along the time axis [58].

Finally, since the output pulse energy increases proportionally with *g*_0_, the maximum output pulse power is reached when *g*_0_ = 1.940. By combining this with the pulse repetition frequency inside the cavity, the intracavity power density is calculated to be slightly lower than the experimental value in reference [20]. Therefore, the phenomenon observed in this study is not affected by optical damage. Meanwhile, in a polarization-maintaining fiber cavity, the relative angle between the initial polarization state and the fiber’s principal axes may still introduce additional degrees of freedom in soliton dynamics through nonlinear birefringence modulation [59]. This study employs an initial Gaussian noise pulse (with its polarization direction aligned with the slow axis by default) to focus on the dominant roles of gain, dispersion, and nonlinear effects in soliton formation and bound-state evolution.

## 4. Conclusions

In this work, we establish a physical model for soliton propagation in femtosecond fiber lasers based on Cr_2_S_3_ SA, further revealing the formation and evolution of mode-locked solitons, soliton transition states, and high-order bound solitons. By optimizing the cavity structure to reduce the net dispersion, the pulse width of the mode-locked soliton is reduced to 329 fs, with a corresponding TBP of 0.332. Through the gradual increase of the small-signal gain coefficient *g*_0_, which corresponds to the pump power, the transition from mode-locked solitons to high-order solitons is initially observed. Subsequently, stable double-bound solitons are achieved, with the soliton pair maintaining a dynamic equilibrium with a spacing of approximately 5.17 ps. Finally, a (2 + 1) type triple-bound soliton is realized, consisting of a single soliton and a soliton pair, where the spacing between the soliton pair remains unchanged. As the small-signal gain coefficient is further increased, more soliton splitting is due to the peak power effect [60]. These results demonstrate that Cr_2_S_3_ SA possesses excellent nonlinear properties and holds significant potential as a saturable absorber in ultrafast fiber lasers. This study contributes to the further investigation of the multifunctional characteristics of bound states, offering insights into their interaction mechanisms as well as the design of multifunctional fiber lasers and potential applications in optical information storage and high-capacity optical communication.

## Figures and Tables

**Table 1:** Initial pulse parameters.

Parameter	Value
*λ*_0_/nm	1550
*T*_max_/ps	60
*M*	2^10^
*NL*	0–1
*T*_FWHM_/ps	10
*C*_0_	0

**Table 2:** Fiber parameters.

Fiber Type	*β*_2_/ps^2^/km
EDF	23.495
SMF	−22.86

## Data Availability

The original contributions presented in the study are included in the article, further inquiries can be directed to the corresponding authors.
